# Viral Transmission in Sea Food Systems: Strategies for Control and Emerging Challenges

**DOI:** 10.3390/foods14061071

**Published:** 2025-03-20

**Authors:** Dingsong Lin, Wendi Chen, Zejia Lin, Lingdai Liu, Molan Zhang, Hongshun Yang, Zifei Liu, Lin Chen

**Affiliations:** 1 Department of Food Science and Technology, Faculty of Science, National University of Singapore, Singapore 117543, Singapore; 2 College of Light Industry and Food Sciences, Zhongkai University of Agriculture and Engineering, Guangzhou 510225, China; 3 School of Chemistry, Chemical Engineering and Biotechnology, Nanyang Technological University, Singapore 637459, Singapore

**Keywords:** aquatic products, food safety, SARS-CoV, seafood, supply chain, virus

## Abstract

The SARS-CoV-2 pandemic had widespread and severe impacts on both the global economy and human health. Facing the continuously mutating virus, this crisis has heightened concerns among consumers and businesses regarding viral transmission through seafood, particularly in the face of emerging, unknown viruses, underscoring our preparedness gaps. This review provides a succinct overview of the survival mechanisms of prevalent viruses in seafood, examines potential transmission pathways to humans during seafood processing, and discusses strategies for mitigating their spread throughout the seafood supply chain. Furthermore, the discussion highlights emerging trends in innovative antiviral technologies aimed at enhancing food safety. Person-to-person transmission remains the most likely source of infection within the supply chain. Therefore, it is still imperative to adhere to the implementation of standard processes, namely good manufacturing practices (GMP) and good hygiene practices (GHP), in the seafood business. In light of the significant losses caused by this crisis and the persistent presence of various viruses within the seafood supply chain, efforts are needed to implement predictive and preventive measures against potential emerging viruses. Future research should focus on monitoring and limiting viral transmission by integrating Industry 4.0 applications, smart technologies, and antiviral packaging, maximizing the potential of these emerging solutions.

## 1. Introduction

Fisheries and aquaculture have a growing significance in the provision of food, nutrition, and employment. In 2022, global aquaculture production reached 130.9 million tons, the highest level in history, representing a 6.6% increase compared to 2020 [1]. In recent years, global consumption of aquatic foods has markedly expanded and is anticipated to persist in its ascent. By 2030, the production of aquatic animals is projected to grow by an additional 14%. Aquatic food systems sustain the lives and livelihoods of millions of people [2].

Seafood spoilage and deterioration pose significant threats, leading to the loss of valuable components and causing food safety concerns. Microbial and viral contaminations are significant contributors to deterioration and pose risks to consumer health. Seafood products often serve as vectors for the transmission of viruses, such as norovirus, rotavirus, Aichi virus, hepatitis A virus, hepatitis E virus, sapovirus, adenovirus, and enterovirus [3,4]. Li [5] reported that seafood products are highly susceptible to contamination by norovirus, and effective measures to control its outbreak have yet to be developed. Hepatitis A virus is one of the most severe viruses associated with shellfish products [6]. Shellfish are filter-feeding organisms, enabling them to amass significant amounts of viruses and bacteria in their consumable tissues. Oysters can concentrate hepatitis A virus (HAV) 100-fold from fecally contaminated water [7]. Noroviruses (NoVs) have been demonstrated to bind to carbohydrates present in the gastrointestinal cells of oysters, clams, and mussels [8]. Viruses remain stable in the environment and are transmitted through the consumption of contaminated water or food or through contact with contaminated surfaces. These viruses can cause a variety of diseases in different individuals and may be challenging to associate with common epidemic causes [9].

Traditional heat inactivation methods have limited effectiveness against these viruses, and since seafood is often consumed raw in the supply chain, heat inactivation is inappropriate. Research indicates that hepatitis A virus (HAV) can survive at 60 °C for one hour, and requires exposure to 98 °C for one minute to be completely inactivated [10]. Norovirus (NoV) retains its infectivity after being heated at 60 °C for 30 min [11]. Depuration methods are also ineffective. Polo [12] found that depuration for hepatitis A virus removal from mussels resulted in an average reduction in HAV levels of 1.1 log units. However, the average final viral loads in shellfish samples remained at relatively high levels and were still infectious. Conversely, atmospheric pressure plasma (APP) jet and gamma irradiation have shown promising results. Kang [13] mentioned that 3–10 kGy of gamma irradiation can effectively reduce the content of norovirus in seafood. Huang [14] found that APP jet treatments could be implemented as an effective technology for the inactivation of food-borne viruses and that it exhibited high potential for application in salmon sashimi processing, retaining product quality as well.

Alongside conventional seafood-borne viruses, novel threats have arisen. In the past, our focus was solely on the viruses transmitted by seafood itself, without recognizing that respiratory diseases could also spread through seafood products. Regarding coronaviruses, SARS-CoV and MERS-CoV were transmitted in 2003 and 2012, respectively [15]. However, as these were regional transmissions, there were no reports of impacts on the seafood industry. The recent SARS-CoV-2 pandemic has inflicted devastating impacts on the global economy and public health. Throughout this global epidemic, both consumers and the industry voiced considerable apprehension and challenges pertaining to the safety and security of seafood. In many countries, transportation restrictions, particularly during the initial months of the pandemic, completely disrupted fisheries and aquaculture supply chains.

Many factories, markets, and restaurants were compelled to close, and fishing and aquaculture activities were halted, nearly interrupting the fragile seafood supply chain [16]. The international community has accepted the reality of coexisting with SARS-CoV-2; however, concerns about the virus’s exponentially increasing mutation rate are mounting. Manufacturers and processors resuming production may face the risk of coronavirus transmission. For example, in 2020, the United States Centers for Disease Control and Prevention (CDC) reported over 100 instances of SARS-CoV-2 cross-infections and fatalities in food processing plants [17]. In the first few months of the pandemic alone, 13 outbreaks occurred in seafood processing facilities and fishing vessels in Alaska [18]. On the other hand, food safety issues represent another significant impact of SARS-CoV-2 on the seafood sector. Given the perishability of seafood, freezing is a common technique for preserving fresh seafood. Nonetheless, a growing number of positive detections related to the seafood cold chain indicate that the coronavirus may endure in refrigerated or frozen environments [19,20]. Additionally, the coronavirus has been shown to survive for several days on surfaces such as stainless steel, plastic, and other materials [21].

Outbreaks related to cold chain seafood products have undermined public confidence in food safety and are expected to further affect the demand. Despite all countries opting to coexist with SARS-CoV-2 and enter the post-pandemic era, it is crucial to recognize the vulnerability of seafood safety amid global viral pandemics. Therefore, it is essential for the seafood industry to clearly understand the transmission pathways of various seafood-related viruses. This understanding will enable the implementation of appropriate preventive measures to prevent the recurrence of viruses, such as the current coronavirus, within the supply chain. Additionally, it is crucial to communicate the safety of seafood products to consumers based on the current evidence. This review aims to identify the primary threats posed by various viruses to the internal operations and supply chains of the seafood industry by compiling available public information and scientific opinions. Additionally, it seeks to provide control measures and best practices to ensure food safety and supply chain continuity in the post-pandemic era, and to prepare for future invasions by new mutated viruses or other potential viruses.

## 2. Transmission Pathways of Viruses in the Seafood Supply Chain

Viruses are stable in the environment and can be transmitted via the ingestion of contaminated food or water, or through contact with contaminated surfaces. Viral infections associated with seafood primarily arise from viruses that accumulate in marine environments and from surface contamination along the seafood supply chain. Therefore, understanding the main viruses disseminated by seafood and their transmission methods allows for better strategies to address viral outbreaks in the seafood supply chain.

### 2.1. Viruses in Seafood

Viruses are small acellular structures with diameters ranging from 20 to 400 nanometers [22]. One milliliter of seawater may harbor millions of virus-like particles that demonstrate pathological activity across all biological taxa, including humans, leading to various diseases [9]. Due to the structure of viruses, their survival duration in the environment can be extended. Pathogenic viruses in filter-feeding shellfish can accumulate more than in the surrounding seawater. Certain migratory shellfish, such as lobsters and crabs, can acquire viruses from contaminated seawater, migrate to cleaner waters, and subsequently act as vectors for pathogenic viruses [9]. Following the SARS-CoV-2 epidemic, the U.S. Centers for Disease Control and Prevention (CDC) reported cross-infections and fatalities related to SARS-CoV-2 in over 100 food processing plants [17]. To this end, the coronavirus, as a novel viral invader in the seafood supply chain, signifies that other similar respiratory transmitted diseases could potentially have the same impact. Research indicates that coronaviruses can persist on stainless steel, plastic, and several other surfaces for multiple days [23], leading to widespread surface contamination. Viruses, whether originating from seafood or transferred via surface contamination in the supply chain, present considerable issues within the seafood supply chain. Table 1 summarizes common food-borne viruses found in seafood, their virus families, associated food commodities, and the clinical diseases they cause. Viruses such as hepatitis A virus (HAV), norovirus (NoV), sapovirus, enterovirus, hepatitis E virus (HEV), astrovirus, human parvovirus, human adenovirus (HAdv), hantavirus, and Aichi virus are listed. Most of these viruses are linked to bivalve molluscan shellfish, contaminated water, and other food sources such as fresh produce, salad, raw or undercooked meat, and unpasteurized milk. The diseases caused by these viruses range from gastroenteritis and hepatitis to neurological symptoms and respiratory illnesses like hantavirus pulmonary syndrome [24].

#### 2.1.1. Norovirus

Norovirus is a highly contagious, non-enveloped, single-stranded RNA virus with a diameter of 28 to 35 nanometers. Norovirus infections are considered as the leading cause of non-bacterial gastroenteritis, frequently linked to the consumption of raw or undercooked shellfish, particularly oysters, or exposure to contaminated water [26]. Infected seafood handlers are potential sources of sporadic cases and outbreaks. Cultivating fish and shellfish in areas near sewage-contaminated waters heightens the risks of pathogen transmission to humans. Mussels, oysters, clams, crabs, shrimp, fish, and blue crabs are the principal carriers of norovirus due to their filter-feeding behavior, allowing them to concentrate viruses from polluted water [26]. Norovirus has been detected in various seafood samples, particularly in oysters, sea urchins, mussels, and semi-dried seafood products. In oyster samples from the French market, NoV viral loads ranged from 10^3^ to 10^6^ genome copies/g [27]. In sea urchin samples from the Portuguese market, NoV viral loads ranged from 8.7 × 10^3^ to 3.2 × 10^4^ genome copies/g [28]. Additionally, semi-dried seafood products that were not heat-treated may carry norovirus [13]. In the United States and the European Union, 50% of foodborne gastroenteritis is caused by norovirus infections [29]. Norovirus is resistant to freezing but sensitive to autoclaving. Nonetheless, it maintains stability in aquatic environments and shellfish, enduring for up to 7 days in purified shellfish [30]. Also, norovirus can survive outside the host, is resistant to common disinfectants and significant pH fluctuations, and is highly contagious. Consequently, the virus is probably transmitted through contaminants. Norovirus spreads among individuals via contaminated food, water, or fecal–oral routes and the environment. Numerous foodborne norovirus outbreaks have been reported, often attributed to infected food handlers [9]. Currently, there is no vaccine available against norovirus, and with current technology, it is difficult to eradicate enteric viruses from live shellfish [31]. The primary strategy involves depurating shellfish; however, although depuration can reduce the number of *E. coli*, it struggles to prevent norovirus infections. Norovirus inactivation methods mainly include high-pressure processing (HPP), heat treatment, chlorine disinfection, and UV treatment. HPP at 300–450 MPa can reduce 2.8–4.0 log of viral load, but even at 600 MPa, complete inactivation may not be achieved, as human challenge studies suggest that lower doses can still cause infections [32]. Heat treatment at 90 °C for 90 s can reduce the viral load by 3 log, while 80 °C for 5 min can inactivate >99.99% of norovirus [33]. Chlorine disinfection at 2.5 mg/L for 60 min achieves >3 log viral reduction [34]. UV treatment at 195–269 J/m^2^ results in a 99.99% (4 log) viral reduction [33]. As a result, beyond the direct impact on health, norovirus outbreaks also damage the seafood supply chain and affect public confidence in these products [35]. Additionally, studies have found significant differences in the uptake and removal rates of viruses among different shellfish species, which complicates the formulation of effective depuration strategies [12]. Therefore, there is a need for more universally effective new technologies to reduce the likelihood of norovirus transmission through seafood, thereby minimizing its impact on the seafood supply chain.

#### 2.1.2. Hepatitis A Virus (HAV)

Hepatitis A virus (HAV) belongs to the Hepatovirus genus of the family Picornaviridae, order Picornavirales, and class Hepatoviridae [36]. Hepatitis A is a globally prevalent disease, with approximately 1.4 million cases reported annually, 50% of which occur in Asia [37]. In 1988, China experienced its largest hepatitis A outbreak, involving raw clams as the seafood source. This outbreak led to 292,301 infections and 47 deaths [38]. From 1986 to 2012, 46 HAV outbreaks were reported globally, linked to seafood carriers such as oysters, clams, mussels, and cockles [39], primarily due to shellfish concentrating viruses from contaminated seawater. In seafood samples, HAV has primarily been detected in oysters, clams, and mussels. In Taiwan’s oyster farming waters and oyster samples, HAV was detected, though no specific viral loads were reported [40]. In a 2008 outbreak, clams were found to contain HAV, with viral loads ranging from 5.1 × 10^3^ to 1.2 × 10^5^ genome copies/g [40]. HAV was also detected in mussel samples collected from the Norwegian coast, indicating possible marine contamination [41]. Humans are the only hosts, and transmission generally occurs through the fecal–oral route. Food can become contaminated through infected individuals’ fecal matter or via contaminated water, which is common in shellfish. Hepatitis A is one of the more severe foodborne diseases, particularly among viral infections. Despite HAV’s inability to proliferate in the environment, it is regarded as highly resilient to diverse environmental conditions, such as freezing, elevated temperatures, chemical exposure, and desiccation [9]. Currently, a vaccine is available for the Hepatitis A virus (HAV) [42]; However, there are no specific therapies or treatments for acute infections caused by HAV or HEV [43]. Like norovirus, using depuration to reduce viral loads and maintaining proper hygiene and personal health practices during the food handling stage are crucial in controlling the spread of hepatitis viruses [44]. Currently, the main strategy is to urge consumers to avoid consuming uncooked shellfish to reduce HAV infections, but there are no effective methods at the supply chain stage. Hepatitis A virus (HAV) inactivation methods include high-pressure processing, heat treatment, and chlorine disinfection. For example, 400 MPa high-pressure processing for 1 min can reduce the viral load by 3 log [32]. Heat treatment is the most effective method for HAV inactivation, with 70 °C for 2 min reducing 3.5 log, and 80 °C for 1 min reducing >3.5 log [33]. Additionally, chlorine disinfection at 2.5 mg/L for 60 min significantly reduces HAV viral load, achieving >3 log inactivation [34].

The high resistance of these viruses to different environmental conditions and physical factors gives them a strong potential for transmission through food [45]. Therefore, in addition to urging workers to get vaccinated, we also need new technologies to reduce HAV contamination in food preparation areas to decrease the occurrence of diseases associated with the virus and protect the supply chain. To achieve this, methods are particularly needed to control and remove HAV from contaminated surfaces [46].

#### 2.1.3. Hepatitis E Virus (HEV)

Hepatitis E virus (HEV) is classified under the Hepeviridae family within the order Hepevirales, genus Orthohepevirus [47]. The first reported case of HEV was related to the consumption of undercooked shellfish by a Japanese patient returning from Vietnam in 2003 [48]. During a global cruise, shellfish (shrimp, lobster, crab, mussels, and scallops) and mixed seafood (a combination of shrimp, salmon, cod, mussels, cod, and squid) caused acute HEV infection in 33 participants [49]. HEV has also been detected in other aquatic organisms, including dolphins, which display clinical symptoms of HEV infection [50]. The contamination of fish and other food sources might be the cause, underscoring the necessity of examining the presence of HEV in these animals to ascertain whether fish consumption poses a risk of HEV infection in people. HEV is primarily transmitted through the fecal–oral route, with contaminated water and raw or undercooked animal-derived foods serving as the primary sources of infection [51]. Hepatitis E virus (HEV) has been detected in oyster samples, particularly along the Norwegian coast [41]. In bivalve shellfish samples from Hebei Province, HEV viral loads ranged from 3312 to 20,350 copies/2 g [52]. Accurate and early identification of HEV in food is crucial for successfully managing outbreaks, implementing preventive measures, and conducting public health interventions [45]. At present, one hepatitis E vaccine, HEV 239 vaccine (Hecolin^®^), has been commercially developed and licensed in China, and has demonstrated high efficacy against hepatitis E [53]. HEV shares many similarities with HAV, thus strategies applicable to HAV are also suitable for HEV. Hepatitis E virus (HEV) inactivation methods primarily include heat treatment and UV disinfection. Studies show that heating at 80 °C for 1 min or 70 °C for 2 min can reduce HEV by 3.5 log [33]. Boiling at 71 °C for 5 min completely inactivates HEV, with no detectable residual infectivity in animal studies [33]. UV disinfection is also effective, as 195–269 J/m^2^ UV irradiation can reduce HEV by 99.99% (4 log) [33].

However, while there are standardized testing methods for HAV (ISO), there is still no standardized protocol for detecting HEV in food. Many groups are currently working to develop a reliable and agreed-upon methodology that incorporates all necessary criteria [45]. Increasing the frequency of HEV testing and standardizing detection methods at various stages of the supply chain can effectively control its spread within the chain, thereby reducing the impact of viral outbreaks on the supply chain.

#### 2.1.4. Human Adenovirus (HAdV)

Human adenoviruses (HAdVs) constitute a notable category of enteric DNA viruses that cause human infections [54]. HAdVs are foodborne enteric pathogens transmitted via the ingestion of contaminated shellfish, commonly detected in seafood [4]. A study conducted in Mumbai markets found adenovirus DNA in 21.27% of all analyzed seafood samples, with the highest incidence in clams (14.89%), followed by oysters, shrimp, and finfish (each 2.13%). These viruses are generally identified in water bodies, including marine environments and rivers [55], largely due to pollution from urban runoff, point-source discharges, and the disposal of fecal pollutants from ships. Shellfish farms, often located nearshore and close to pollution points, are prone to harboring adenoviruses [56]. Adenovirus has primarily been detected in oysters and mussels. In Taiwan’s oyster farms and fishing ports, adenovirus loads ranged from 1.23 × 10^3^ to 1.00 × 10^6^ copies/L (seawater) and 3.57 × 10^3^ to 4.27 × 10^4^ copies/100 g (oysters) [57]. Adenovirus was also detected in mussel samples from the Norwegian coast, suggesting possible human fecal contamination [41]. Furthermore, insufficiently treated sewage discharged into coastal areas might contaminate fishing ports, resulting in adenovirus infections in the caught fish. Poor sanitation conditions and inadequate hygiene facilities in fishing ports may also contribute to seafood contamination, facilitating the transmission of adenovirus via seafood consumption [56]. Adenoviruses can be transmitted through droplet infection, fecal–oral routes, and foodborne zoonosis [58]. These viruses are highly resistant to traditional sewage treatment systems and can survive in harsh environments with high survival efficiency [55]. The transmission outbreaks of these viruses are persistent and widespread, as reported in Egypt [59], the United States [60], and China [61]. Currently, a vaccine against this virus is only available for US military personnel [62], and is not accessible to the general public, thus making it difficult to reduce transmission through vaccination of workers. Adenoviruses can survive in water for extended periods, so efficiently separating and purifying wastewater within the supply chain is key to reducing the risk of adenovirus infections. Chlorine inactivation is considered a viable method [63]. Adenovirus (AdV) inactivation primarily relies on chlorine disinfection. For example, 2.5 mg/L chlorine for 60 min significantly reduces adenovirus load, demonstrating a strong inactivation effect [34]. Therefore, effective and timely testing and proper treatment of wastewater at various stages of the supply chain can significantly reduce the risk of spreading adenoviruses through the supply chain, minimizing losses.

#### 2.1.5. Coronavirus (CoV)

Coronaviruses are enveloped single-stranded RNA viruses that can be categorized into four major groups based on virology and genetics: α-coronaviruses, β-coronaviruses, γ-coronaviruses, and δ-coronaviruses [64]. Seven coronaviruses have been recognized as capable of infecting humans and spreading interpersonally. In contrast, severe acute respiratory syndrome coronavirus (SARS-CoV) and Middle East respiratory syndrome ccoronavirus (MERS-CoV) precipitated lethal widespread outbreaks in 2003 and 2012, respectively.

**Figure 1:**
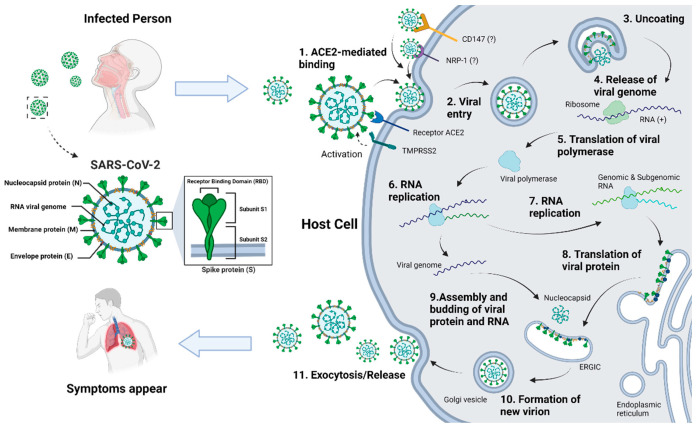
The general schematic representation of the structure, life cycle, and infection mechanism in human cells of SARS-CoV-2. (Note: Created with BioRender.com. Partially adapted from “Coronavirus Structure and Protein Visualization”, by BioRender.com (2021). Retrieved from https://app.biorender.com/biorender-templates [access on 17 December 2021]). (?) The role of CD147 and NRP1 as direct receptors in the SARS-CoV-2 infection pathway remains controversial and lacks definitive experimental evidence.

SARS-CoV-2 belongs to the genus β-coronavirus. The structure of SARS-CoV-2 includes four structural proteins: nucleocapsid protein (N), membrane protein (M), spike protein (S), and envelope protein (E), as well as an RNA genome strand (Figure 1). The spike and membrane proteins on the virus surface form a crown-like appearance, playing a key role in recognizing and invading cells [65]. Additionally, the spike protein confers high transmissibility to the virus. Multiple studies have demonstrated that the receptor-binding domain (RBD) on the S1 subunit of the spike protein can recognize and bind to the angiotensin-converting enzyme II (ACE2) receptor on host cells, thus facilitating the entry of SARS-CoV-2 into host cells [64,65,66]. As the functional receptor for SARS-CoV-2, ACE2 receptors are widely expressed in human alveolar and small intestine epithelial cells [67]. The prevailing view regarding the fundamental route of viral entry into cells is that airborne SARS-CoV-2 infiltrates the human respiratory tract and attaches to the ACE2 receptors on lung epithelial cells (Figure 1) [66]. Subsequently, transmembrane serine protease 2 (TMPRSS2) assists in the fusion of the virus with host cells through endocytosis, resulting in the release of viral single-stranded RNA [66]. Furthermore, two recently proposed protein receptors, neuropilin-1 (NRP-1) and CD147, may serve as new potential pathways for SARS-CoV-2 entry into cells; nevertheless, further exploration of their reliability is needed [68,69].

After entering the cell, the virus unpacks and releases RNA, subsequently employing the host cell’s nuclear translation to produce the required viral polymerase for RNA replication (Figure 1). Viral structural and non-structural proteins are synthesized and replicated with the help of the replication–transcription complex [66]. Ultimately, the translated structural proteins assemble with the new genome in the endoplasmic reticulum–Golgi intermediate compartment (ERGIC) and await release into the extracellular space to infect new cells (Figure 1). At this point, the infected individual progressively develops symptoms such as cough, fever, and anosmia.

Due to the extensive scope of the coronavirus outbreak, its impact on the seafood supply chain is more severe compared to traditional viruses. Traditional viruses typically cause localized, small-scale effects on specific parts of the supply chain; however, coronaviruses like SARS-CoV-2 cause disruptions to production, labor, distribution, supply, and demand and create a range of impacts [70]. Typically, traditional viruses are transmitted through seafood itself, which makes detection and monitoring more targeted. However, the source of coronavirus is highly uncertain, and any worker at any part of the supply chain could potentially introduce and spread the virus throughout the entire chain. Additionally, the presence of human coronaviruses in foods is drastically understudied, and no records on the presence of seasonal and endemic HCoVs in foods could be found in the literature [71]. Therefore, we can only implement appropriate measures and employ new technologies to reduce the impact of such respiratory diseases on the supply chain based on the available information.

### 2.2. Transmission Routes

The persistence of viruses is frequently a pivotal aspect in ascertaining their transmission pathways. Environmental conditions, such as temperature, pH, and humidity, significantly affect the stability of viruses [23]. For example, SARS-CoV-2 has prolonged viability on salmon surfaces at 4 °C compared to 25 °C, lasting for up to a week [72]. At room temperature, SARS-CoV-2 exhibits exceptional stability across a pH range of 3 to 10 [73]. In open seawater, norovirus can still be detected after three to four weeks of purification [74,75], and simply heating shellfish to open does not eliminate norovirus due to its thermal stability. HAV shows considerable environmental resilience and may survive in both freshwater and saltwater habitats for up to 12 months [76]. These finding indicate that food factories and markets associated with cold chain processing may provide environments conductive to virus survival. Therefore, delineating the transmission routes of these viruses throughout the seafood supply chain is a prerequisite for developing effective control measures in the seafood industry. The primary transmission route of coronaviruses is person to person, but there remains contention on the potential for alternative transmission pathways to disseminate the virus. Norovirus spreads among individuals via contaminated food, water, or fecal–oral routes and environmental exposure, while HAV and HEV primarily propagate through the fecal–oral route.

#### 2.2.1. Respiratory Transmission

**Figure 2:**
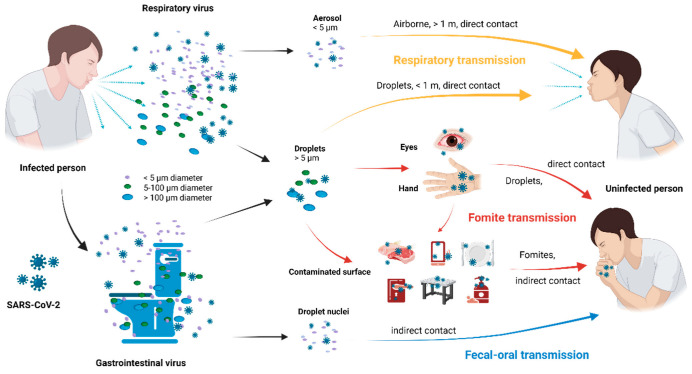
The main proposed transmission route of SARS-CoV-2. (Note: Created with BioRender.com).

For most of the last century, respiratory viruses were believed to be primarily transmitted through larger respiratory droplets. Droplets are produced when infected individuals cough or sneeze, and they deposit on the mucous membranes of potential hosts’ eyes, noses, or mouths (droplet transmission) or on surfaces that potential hosts subsequently touch and then transfer these pathogens to their mucous membranes (contaminant transmission) [77]. Before 2019, respiratory transmission was rarely linked to seafood-borne viruses until the detection of SARS-CoV-2 at a seafood market in Wuhan, marking the first foodborne outbreak transmitted through respiratory routes [78]. As the current pandemic’s focal point, SARS-CoV-2 is undoubtedly the respiratory-transmitted virus that poses the most significant potential threat to the seafood supply chain. The respiratory transmission of SARS-CoV-2 can occur through droplets or aerosols (Figure 2, represented by yellow arrows). SARS-CoV-2 is primarily spread interpersonally via droplets larger than 5 μm generated by coughing, sneezing, and respiration [79]. Due to gravity, these respiratory secretions quickly settle on surfaces or the ground within 1 to 2 m. Another possible transmission route is aerosol transmission [80]. Aerosols are tiny particles (under 5 μm in diameter) produced by regular respiration, talking, or the evaporation of respiratory droplets. Aerosols can persist in the air for extended periods and traverse longer distances [81]. Van Doremalen [21] found that SARS-CoV-2 could survive in aerosols for up to 3 h. Additionally, aerosols are very susceptible to air currents and can disperse over extensive distances. However, current scientific knowledge suggests that adhering to social distancing of 2 m or more, wearing masks, and maintaining proper personal hygiene can effectively mitigate most droplets and airborne transmission [82].

Furthermore, while no evidence has proven that adenoviruses and enteric viruses commonly found in seafood impact the seafood supply chain through respiratory transmission, it has been established that they can be transmitted through respiratory routes [83]. As an enteric virus, for norovirus, although there is no evidence that it spreads through the air [84], several publications have highlighted the significance of this route [85]. After SARS-CoV-2, we should evaluate whether previous viruses may have possessed alternative transmission routes in the seafood supply chain.

Crowded and confined indoor spaces are crucial factors that increase the risk of airborne transmission [86]. In enclosed environments with poor air circulation, droplets and aerosols are more likely to accumulate and remain suspended for longer periods, thereby increasing the risk of infection [87]. In the seafood supply chain, congested locations such as factories, restaurants, and retail markets can be considered as enclosed and efficient transmission sites. For example, a report from Guangzhou, China, showed that the trajectories of 10 cases of SARS-CoV-2 infection in an enclosed restaurant aligned consistently with the airflow path generated by air conditioning systems in the contained space [19]. Ensuring well-ventilated workspaces or switching to open areas can reduce the risk of indoor cluster transmission.

#### 2.2.2. Contact Transmission

While pathogens sometimes spread through direct contact between infected and vulnerable individuals, the environment is often a crucial medium for transmission. Water, food, and contaminants can act as environmental reservoirs, enhancing the ability of pathogens to spread among hosts [88]. Object transmission is a type of contact transmission which is defined as the spread of pathogens through a contaminated intermediate object [89]. The study by Clay [90] suggests that both non-enveloped and enveloped viruses, such as norovirus, can survive on environmental contaminants for hours to days, a period long enough to infect susceptible individuals. Mbithi [91] assessed the survival rate of hepatitis A virus (HAV) on human hands, revealing that 16% to 30% of the virus could be recovered from hands after 1 h of drying. The study by Abad [92] showed that HAV and adenovirus can survive on several commonly used materials in household environments for extended periods, although the duration varies depending on the specific substance. Shellfish, which can concentrate potential viruses from seawater, easily contaminate the seafood supply chain with viruses such as HAV, HBV, norovirus, and adenovirus. Accordingly, workers handling these viruses may inadvertently spread them to packaging or other products on the production line, thereby heightening the risk of outbreak propagation.

Similarly, in the context of SARS-CoV-2, contact transmission is as common a mode as airborne and aerosol transmission (Figure 2, represented by red arrows [80,93]. The virus-laden droplets settle from the atmosphere onto surfaces, resulting in contamination. Like most human viruses, although SARS-CoV-2 can survive outside the body for long periods, it cannot replicate without a host [94]. Therefore, after contacting these contaminated surfaces, the virus may spread through human hands to the mouth, nose, or eyes, leading to indirect infection. Potential contaminants may include work surfaces, utensils, food packaging, or the food itself. Several studies have summarized the survival capabilities of SARS-CoV-2 on different food packaging materials (Table 2) [21,95]. This table presents the survivability of SARS-CoV-2 on various material surfaces at room temperature. The virus remains viable for different durations depending on the surface type. It can persist for up to 3–4 h in aerosols, while on plastic and stainless steel, it survives for up to 72 h. On copper, the viability is limited to 4 h, whereas on cardboard and cloth, it lasts up to 24 h. The virus remains detectable for up to 96 h on glass and surgical masks and up to 48 h on steel. These findings highlight the varying stability of SARS-CoV-2 on different materials, which is crucial for understanding transmission risks. Additionally, SARS-CoV-2’s infectiousness significantly remains on various material surfaces in the presence of proteins [94]. Work surfaces or food packaging in places such as fishing boats, seafood processing plants, retail markets, and restaurants may not only become potential high-risk contamination sites but also extend the infectivity of SARS-CoV-2 by introducing food residues.

Food safety is an unavoidable topic for the seafood industry. Contaminants from upstream in the seafood supply chain may spread throughout the supply chain through transportation and global trade. One piece of evidence is the detection of SARS-CoV-2 remnants on imported salmon at Beijing’s Xin fadi market, which was considered as the source of another outbreak in Beijing in June 2020 [19]. The Chinese government tested imported frozen salmon samples and found several positive viral results. Additionally, two dockworkers in Qingdao, China, who had no history of contact with SARS-CoV-2 patients, were believed to have been excessively exposed to frozen cod packaging that tested positive for SARS-CoV-2 RNA [19]. This has triggered public panic and concerns about food safety, cross-border trade, and frozen food products [20]. This may indicate that contaminants can spread over long distances under low-temperature conditions. On the other hand, frozen seafood and packaging materials may act as carriers for virus transmission throughout the supply chain.

These findings indicated that coronaviruses may spread along the supply chain by attaching to food packaging and frozen seafood. However, it is essential to recognize that there is still a lack of direct and compelling evidence regarding the transmission of SARS-CoV-2 from objects, including food packaging and frozen food [19]. Some scholars question whether investigations of cold chain products overlook the possibility that retail workers might have acquired the virus from international traders or asymptomatic individuals [96]. The mobility of workers complicates the screening of numerous asymptomatic patients in international trade. The World Health Organization has also pointed out that while object transmission is theoretically feasible, pathogenicity requires sufficient live viruses on the object.

Undoubtedly, human respiratory droplets are the primary carriers and sources of objects. Wearing a mask properly is adequate to effectively reduce the transmission of various viruses [96]. Manufacturers and supply chain managers should also choose appropriate surface disinfectants based on the nature of the work and the surface materials to prevent the spread of harmful objects. The World Health Organization recommends the use of hand sanitizers and 70–90% alcohol as effective surface disinfectants [97]. Ultraviolet (UV) disinfection is also an efficacious method for inactivating numerous viruses and is currently used to sanitize SARS-CoV-2 on imported packaging surfaces [74]. Lu [19] and Chin [73] summarized several surface disinfectants that effectively inactivate SARS-CoV-2, with some results outlined in Table 3. This table outlines various disinfectants and methods effective in inactivating SARS-CoV-2, along with their working concentrations, treatment times, and virus reduction levels. Heat treatment at >75 °C for 45 s to 5 min or 70 °C for 5 min significantly reduces the virus. Chemical disinfectants, including sodium hypochlorite (0.05%), ethanol (70%), povidone-iodine (7.5%), chloroxylenol (0.05%), chlorhexidine (0.05%), and benzalkonium chloride (0.1%), are effective, with reductions of over 99.9% (3.8 to 5.8 log reduction) after 5 min. Silicon nitride suspensions (15 wt%) inactivate 99% of the virus in 1 min, while ozone (4 ppm gas exposure for 90 min) reduces viral titers by over 98.2%. Household bleach (1:49 and 1:99 aqueous solutions) is also highly effective. These findings highlight the importance of heat treatment and chemical disinfectants in decontaminating surfaces and preventing virus transmission.

In addition to surface disinfection, consumers and workers must wash their hands properly after touching the potentially contaminated surfaces, especially before eating or touching their faces. Additionally, the virus may remain on surgical masks for up to four days [95]. Besides maintaining good personal hygiene and regular cleaning of work surfaces, workers in the seafood supply chain should also be aware of the need to change masks promptly.

#### 2.2.3. Fecal–Oral Transmission and Foodborne Transmission

Viruses depend on living cells for replication and usually only infect a limited range of hosts. Therefore, human viruses generally cannot reproduce in seafood but may contaminate it. The issue of viruses in seafood primarily concerns their passive transmission to humans, particularly those spread via the fecal–oral route, such as gastrointestinal viruses, including hepatitis A and poliovirus [101]. In the case of HAV, humans are the only hosts, with transmission generally occurring via the fecal–oral route. Food becomes contaminated through contact with feces from infected individuals or through fecal-contaminated water, which frequently occurs with shellfish. Hepatitis A is one of the more severe foodborne diseases, particularly among viral infections. Although HAV cannot grow in the environment, it is regarded as highly stable under various environmental circumstances, allowing it likely to persist until it reaches humans and induces infection [9]. Likewise, HEV and norovirus propagate by fecal–oral transmission and foodborne transmission. Fecal–oral transmission and foodborne transmission were the predominant sources of most reported seafood-related viral infections prior to the emergence of SARS-CoV-2.

However, for SARS-CoV-2, fecal–oral transmission is considered as a potentially controversial transmission route (Figure 2, represented by blue arrows). It is still uncertain whether SARS-CoV-2 can cause gastrointestinal infection in humans via the fecal–oral route [102]. Undoubtedly, SARS-CoV-2 can be present in other body fluids, including saliva, urine, feces, semen, and tears, in addition to respiratory secretions [80]. Evidence shows that many SARS-CoV-2 cases present with gastrointestinal symptoms such as diarrhea [103]. Another study suggested that even when nasopharyngeal or oropharyngeal swabs tests are negative, viral excretion can still be detected in the feces of recovered SARS-CoV-2 patients [104]. There is compelling evidence that ACE2 receptors are highly expressed in oral and small intestinal epithelial cells, providing a portal for SARS-CoV-2 to infiltrate the human body and cause infection [105]. Two in vitro studies have shown that SARS-CoV-2 can replicate in intestinal epithelial cells [106]. Therefore, the fecal–oral route should be considered as a potential transmission route [93].

For the labor-intensive seafood industry, communal restrooms may pose a risk of viral transmission. Furthermore, feces or urine excreted by patients may enter wastewater through drainage systems, particularly in poorly maintained factories or farms, where untreated wastewater could pose a potential risk for SARS-CoV-2 transmission [107]. Thus, it is essential to implement wastewater testing in factories and aquaculture farms to ensure the safety of workers, aquatic organisms, and products. It is worth noting that viral fragments in wastewater and feces do not necessarily imply that the virus remains infectious. It is unclear whether SARS-CoV-2 can invade intestinal epithelial cells through food media, as does the requisite viral concentration in the human stomach for infection to occur [102]. One hypothesis is that infection via food intake might occur because the virus on these foods enters the respiratory tract through contact or inhalation before entering the digestive tract.

Foodborne viruses cause human illness through the fecal–oral route, with infection primarily occurring in the human digestive tract [102]. Due to their explicit fecal–oral transmission pathways and strong epidemiological associations with contaminated food, foodborne viruses demand the establishment of surveillance systems in large-scale seafood supply chains to detect most viruses. However, there is currently no substantial epidemiological evidence linking coronavirus to the consumption of virus-contaminated food, nor is there evidence that SARS-CoV-2 attached to food can infect the human digestive tract [24,102]. However, recent reports from various countries have identified clusters of COVID-19 infections associated with the fresh meat and seafood industries, raising concerns about the potential transmission of SARS-CoV-2 to humans through food. Additionally, frequent cases of COVID-19 infections have been reported among workers in slaughterhouses and meat processing plants in countries such as Canada, Brazil, Germany, and Ireland [108]. Since SARS-CoV-2 does not replicate in non-living cells, its infectivity remains low unless sufficient infectious SARS-CoV-2 is transmitted via this route [24]. However, the virus can survive for extended periods on frozen surfaces during transportation and export [20,109,110]. Given that most respiratory viruses require a low infectious dose, the survival of small amounts of the virus in globally exported meat and seafood products may contribute to the global transmission and resurgence of SARS-CoV-2 through the cold chain food supply. Similarly, infected workers may also introduce the virus into the animal food chain. Overall, the risk of contracting SARS-CoV-2 from contaminated food or food packaging is deemed low [102,111]. Given its low food safety concern, coronavirus is still regarded as a respiratory virus rather than a foodborne virus.

#### 2.2.4. Zoonotic Transmission

Before SARS-CoV-2, seafood-related zoonotic diseases only involved parasites [112]. Although HAV and HEV have been reported to cause zoonotic transmission, their hosts, apart from humans, are primarily some primate species [113]. Similarly, adenoviruses have also been reported to cross species between humans and non-human primates, confirming them as zoonotic viruses [58]. However, after the outbreak of SARS-CoV-2 at the seafood market in Wuhan, it became apparent that the initial virus might have been transmitted to people via zoonotic transmission.

SARS-CoV-2 has been identified as a zoonotic virus, indicating that zoonosis may be one of the pathways by which coronaviruses infect humans. However, Godoy [24] suggest that aquatic animals are unlikely to act as intermediate hosts for the transmission of zoonotic diseases. Although there is still debate over whether the original virus was transmitted to humans by bats or pangolins, it has been established that mammals such as minks and domestic cats show susceptibility to SARS-CoV-2 and may have been intermediate hosts in this pandemic [24,114]. This is because many mammals, akin to humans, express the ACE2 genome. There is already clear evidence of animal-to-human transmission of SARS-CoV-2 [115]. Additionally, the Omicron variant that emerged in December 2021 has evolved in house mice and likely spread from humans to house mice before re-emerging in humans after extensive evolution [116]. Research has indicated that mutations in the receptor-binding domain (RBD) of the SARS-CoV-2 virus can enhance cross-species transmission between humans and animals [117]. Therefore, as the virus mutates, the zoonotic transmission of SARS-CoV-2 may intensify. However, studies have shown that the ACE2 receptor gene sequence in fish is only 60% similar to that in humans [98]. Another computational analysis also found that ACE2 receptors in fish, reptiles, and amphibians are unlikely to bind to the S protein in SARS-CoV-2 [118]. Due to the lack of favorable environmental conditions and infection targets for viral replication in aquatic animals such as poikilotherms, there is currently no evidence or indications supporting the infection of aquatic animals by SARS-CoV-2 [24]. Although there is evidence that aquatic mammals such as belugas may be at risk of coronavirus infection [119], this raises concerns more related to biodiversity rather than seafood production. Therefore, aquaculture should prioritize the prevention of interpersonal transmission over the spread from aquatic animals to humans.

## 3. Industry Practices and New Technologies for Potential Virus Prevention

### 3.1. Prevention and Control Measures in the Seafood Industry

**Figure 3:**
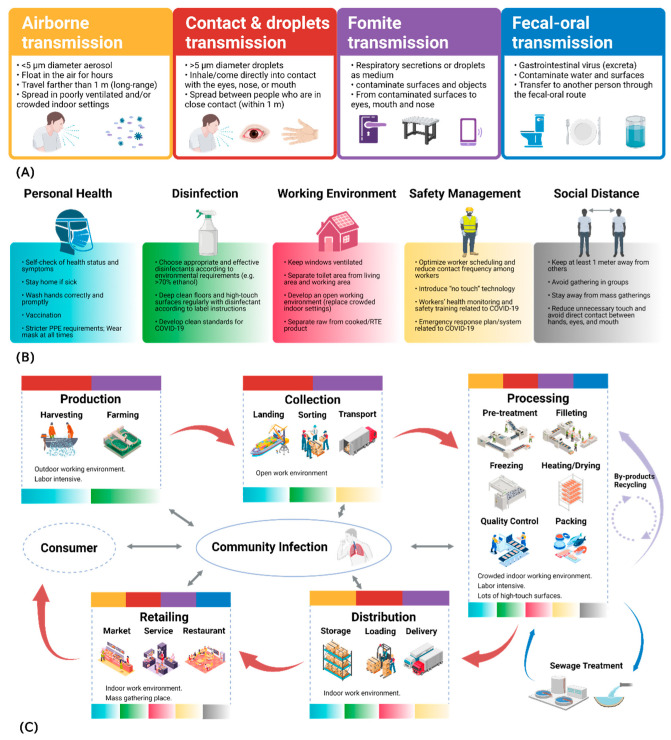
The primary transmission mode of SARS-CoV-2 involved in the seafood supply chain (**A**), proposed safety measures for the seafood industry during the pandemic (**B**), the most common transmission mode (shown at the top of the text box, represented by the solid color in (**A**)) and the most critical safety measures (shown at the bottom of the text box, represented by the gradient color in (**B**)) for each stage of the seafood supply chain from production to consumer (**C**). (Note: Created with BioRender.com).

The procedures used to eliminate the risk of disease transmission by possible viruses in the seafood supply chain are like those employed in the food supply chain. As previously discussed, four transmission routes of seafood-related viruses in the supply chain pose the greatest threat to the seafood industry: airborne transmission, prevalent in settings with poor interior air circulation; droplet transmission, due to close contact; object transmission, arising from contact with contaminated surfaces; and potential fecal–oral transmission (Figure 3A). Although the current coronavirusvirus is not frequently transmitted through food or food packaging materials, following appropriate food safety measures remains crucial to mitigate the risk of other common foodborne diseases caused by more prevalent enteric viruses and human adenoviruses [120]. Figure 3B summarizes the basic preventive measures to halt virus transmission in the supply chain, including personal hygiene, surface disinfection, workplace cleanliness, training and management, and maintaining distance. Figure 3C illustrates the main activities in the fisheries or aquaculture supply chain, the predominant transmission threats at each key stage, and the essential preventive measures. The production, transportation, processing, distribution, and retail marketing stages of fisheries and aquaculture are equally vital to the integrity and success of the supply chain [121]. Each stage is susceptible to different modes of viral transmission, depending on production and environmental factors, which could lead to business disruption and even global food safety issues. Therefore, to safeguard and maintain the sustainability of the supply chain, it is imperative to provide targeted and adequate protection for each stage of the fisheries and aquaculture food supply chain [121].

Sick workers are undoubtedly the greatest potential threat within the aquaculture supply chain. Many common viruses have incubation periods; for instance, hepatitis B extends from 3 to 6 months, while hepatitis A ranges from 2 to 6 weeks. For SARS-CoV-2, the incubation period is 1 to 2 weeks before the onset of symptoms [122], meaning workers at all stages may contract community-acquired infections unexpectedly, leading to outbreak propagation across the supply chain. Working on fishing vessels and engaging in post-harvest handling, packaging, and processing, where the space is constrained and humidity is an issue, increases the risk of viral transmission and outbreaks among workers. Therefore, employee health and safety must be seen as a fundamental preventive precaution across all stages, from production to retail. Recommended measures include employee self-screening for symptoms (fever, cough, shortness of breath, diarrhea) and employer evaluation of employees’ health status [123]. Employers should regularly screen employees based on job types and associated risk factors [79], symptoms, travel history, and whether they have been in contact with coronavirus-related cases or areas prone to norovirus or hepatitis A outbreaks. Additionally, the industry should adopt reasonable sick leave policies to encourage symptomatic employees to rest at home [123]. Hand hygiene effectively prevents the spread of droplets and contact transmission. The FDA recommends that employees wash their hands regularly with effective hand sanitizer and ensure thorough handwashing for over 20 s [124]. Vaccination is a crucial measure in preventing viral transmission. Many countries have included HAV vaccines in national immunization programs, thereby reducing disease incidence and virus transmission rates in outbreak scenarios. Vaccination campaigns should be conducted to mitigate the spread of HAV [125], a method that also applies to the HEV virus. Vaccination is also critical in curbing the spread of SARS-CoV-2 [126]. Research shows that increasing vaccination rates can diminish the risk of virus transmission and effectively lower the rate of viral mutations [127]. The food supply chain plays a crucial role in global food safety, with its integrity directly linked to the health of workers and the sufficiency of food supplies [128]. In countries or regions where vaccine supplies are limited or unevenly distributed, governments ought to prioritize vaccine allocation for high-risk fishery workers, who face greater risks of international travel and virus exposure. Additionally, the proper use of personal protective equipment (PPE) is the last line of defense in decreasing the risk of cross-contamination from viruses and foodborne pathogens [79].

Standard PPE in the seafood industry includes head coverings, face shields, disposable safety gloves, clean work attire, work boots, and, most importantly, masks, which are vital for preventing respiratory transmission. The use of masks can effectively prevent respiratory viruses from infecting healthy individuals and, more importantly, avoid infected workers from inadvertently releasing virus-laden droplets into the air [129]. Although WHO and CDC recommend cloth masks instead of medical masks in cases of shortages of medical N95 and surgical masks [129,130], it is still advised that seafood industry workers continue to wear medical masks whenever possible to maximize protection. Mask surfaces may retain viruses for several days, hence timely mask replacement is necessary [95]. Ideally, a new mask should be replaced each time the mask is removed. However, it is important to emphasize that PPE cannot substitute for other preventive measures. For example, disposable gloves cannot replace handwashing; removing gloves may lead to hand contamination [79]. Surface disinfection is another key measure that should be a focus at all stages, as it effectively prevents object transmission, given that viruses can survive on surfaces such as floors, worktops, and food packaging. Industry cleaning personnel should develop disinfection procedures and regularly and effectively sanitize high-contact areas such as taps, door handles, worktops, and other frequently touched surfaces with disinfectants [131].

While all measures apply to every stage of the supply chain, the risks faced and the significance of executing these measures vary between stages. Unlike aquaculture farms and fisheries, workplaces such as processing, distribution, and retail, typically located in factories, warehouses, restaurants, and markets, present a considerably higher risk of airborne transmission due to crowded indoor settings [86,120]. Therefore, managers should enhance ventilation and air exchange in indoor locations. Food processing areas, such as processing plants and restaurant kitchens, must adhere to basic practices to prevent the spread of foodborne illnesses, such as separating raw and cooked foods [132], especially since raw seafood such as shellfish significantly increases the risk of transmitting viruses like norovirus and hepatitis A. Additionally, because of the potential risk of fecal–oral transmission, toilets should be located away from dining and living areas [86,120]. At the same time, building owners with living areas must ascertain the safety of the building’s drainage system, as there is evidence indicating the risk of viral transmission via plumbing systems [133]. It is advisable that all wastewater treatment and water recycling systems involved in aquaculture production be separate from those of medical institutions to prevent viral-laden wastewater from contaminating natural water bodies used for fisheries and aquaculture activities [134]. Additionally, monitoring viral RNA in wastewater could be one of the most helpful monitoring tools for preventing this pandemic. It can be actively used in public health responses to ensure a safe production environment and food safety [135].

The above methods can effectively reduce viral transmission during the supply chain process. However, it is inevitable that some seafood will carry enteric viruses prior to capture or during cultivation, known as pre-harvest contamination [136]. For norovirus, the predominant viral sources are attributed to the pollution of water bodies where the seafood was caught before harvest; thus, control approaches emphasize the monitoring of fecal coliforms in fishing waters. Over the past 20 years, the reported hepatitis A virus infections associated with seafood consumption in the United States have declined, at least partly due to the implementation of strengthened control measures in the 1980s to prevent fecal contamination of fishing beds. The reduction in outbreaks suggests that this control strategy may effectively reduce the risk of seafood-related infections [137].

In addition to preventive measures against different transmission routes, the industry should strengthen safety management according to actual conditions to address the risk of various viral transmissions within the supply chain:

1. Train employees on skills to prevent the spread of virus, strengthen food safety education, and ensure employees’ mental health [138].
2. Install and provide additional handwashing stations at high-traffic and fixed locations, equipped with warm water and hand sanitizer [79].
3. Establish health screening procedures, such as using quick, non-contact thermometers to check the temperature of every worker or visitor entering the workplace [139].
4. Restrict visitors and personnel movement to minimize unnecessary contact.
5. Provide workers with adequate and clean personal protective equipment (PPE).
6. Introduce labor-saving technologies, such as artificial intelligence [140].
7. Maintain good manufacturing practices (GMP) and good hygiene practices (GHP) [139].
8. Increase water treatment and purification systems at aquaculture farms to filter out viruses and reduce the viral content within seafood products, thus lowering the viral load at the source.
9. Regularly monitor the viral concentration in wastewater produced at each stage of the process and take preemptive measures to investigate and address any abnormalities as soon as they are detected.
10. It is recommended that employees be vaccinated against the relevant viruses.
11. Strengthen the traceability system, so that once a virus is detected, it is possible to promptly determine which stage of the process is problematic and take timely measures to address it.
12. Improve the production line by incorporating commercially viable non-thermal treatments, such as ultraviolet (UV) light, to reduce the quantity of viruses on packaging and products.
13. Adhere to food safety management system (FSMS) protocols established by authorities in accordance with Hazard Analysis and Critical Control Points (HACCP) principles [141,142]. Maintaining social distance above 1 m effectively reduces the probability of airborne virus transmission [143]. However, maintaining social distancing throughout the entire supply chain or within food facilities is complex [124]. For example, most sanitation measures in capture fisheries production complicate fishing activities [121]. Moreover, strengthening port or border restrictions may lead to fishermen remaining at sea for extended periods (due to being unable to land), making physical distancing on fishing vessels less important [28]. Therefore, managers involved in the seafood supply chain should assess operational modifications in implementing physical distancing measures in crowded public facilities such as factories, restaurants, and markets, and adopt optimal strategies to minimize non-work-related interactions [124].

In the seafood supply chain, HACCP is the most comprehensive approach to reducing virus transmission, as it systematically identifies critical control points and mitigates risks before contamination occurs [144]. However, it requires significant investment and technical expertise, limiting its application in small-scale seafood operations [145].

GMP ensures a structured framework for hygiene and sanitation in seafood processing. It has been shown to be effective in preventing viral contamination through controlled production environments and worker hygiene monitoring [144]. However, GMP is less adaptable than HACCP because it lacks continuous hazard assessment and relies heavily on compliance and audits [146].

GHP, while similar to GMP, focuses more on personal hygiene and environmental sanitation. It is highly effective at reducing person-to-product transmission, especially for enteric viruses spread through food handling [146]. Yet, its reliance on individual behavior makes it less reliable than HACCP or GMP, as improper hand hygiene and self-contact behaviors can lead to virus persistence on surfaces [147].

PPE, such as gloves, gowns, and masks, provides immediate protection against viral exposure. However, improper use or doffing errors can lead to the contamination of workers and seafood products [148]. Additionally, PPE does not prevent cross-contamination on surfaces and equipment, making it less effective than systematic approaches like HACCP or GMP [149].

HACCP and GMP require significant financial and technical resources, making them challenging for small seafood processors [145]. GHP is easier to implement but depends on worker compliance [146]. PPE is the most accessible but has the highest risk of misuse [148]. Overall, HACCP is the most effective but costly, GMP is structured but less adaptable, GHP is practical but inconsistent, and PPE is accessible but limited in scope. A combination of all four is ideal for minimizing virus transmission in seafood processing.

Here are some successful cases of control practices effectively reducing seafood-borne diseases. From 1983 to 1993 in the USA, the implementation of HACCP, GMP, and GHP improved seafood processing and cold chain management, reducing seafood-related foodborne illness rates from 10.0% to 7.4% [136]. In 1991, Peru, facing a cholera outbreak, promoted HACCP, GHP, and public health education, strengthening seafood hygiene management and significantly reducing cholera cases over the years [136]. Between 2003 and 2005 in Poland, the adoption of HACCP, GMP, and GHP as part of EU accession requirements led to 91% of factories becoming familiar with GHP and 95% understanding HACCP, greatly enhancing food safety levels [150]. From 1981 to 1990 in Japan, HACCP and freezing treatment (−20 °C for 48 h) were introduced to combat parasitic infections from raw fish consumption, successfully lowering anisakiasis cases [136]. In the UK between 1992 and 1996, strict enforcement of HACCP and GMP regulations in the food industry resulted in a significant reduction of 530 reported food safety incidents [144]. During the 2000s in the USA, the application of HACCP in regulating heat treatment and low-temperature storage of canned seafood successfully minimized botulism and histamine poisoning cases [136]. These cases demonstrate how implementing proper control measures can significantly improve seafood safety and public health.

### 3.2. Policy Implications in the Seafood Industry

Different countries have established varying standards for viral control and processing requirements in the seafood supply chain. The United States follows the FDA’s *National Shellfish Sanitation Program (NSSP)*, primarily focusing on norovirus (NoV) and hepatitis A virus (HAV) detection. UV depuration is a standard bacterial control method but is ineffective against NoV, requiring additional high-pressure processing (HPP) and pulsed light (PL) technology [27]. The European Union (EU) enforces strict *E. coli*-based water quality assessments and mandates depuration, relaying, or heat treatment to eliminate viral contamination [56]. Chinese standard (GB 2733-2015) expands virus monitoring to include rotavirus, while Japan prioritizes raw seafood safety, requiring thorough HAV and NoV testing [151].

For processing, the US and EU enforce HACCP-based cold chain management, requiring quick freezing, high-temperature processing, or depuration [136]. China integrates low-temperature pasteurization, salting, and freezing to mitigate viral risks, while Japan mandates freezing (−20 °C for 7 days) or heating (>60 °C) to eliminate parasites and viruses in raw seafood [151].

While the US and EU impose strict import controls, China and Japan emphasize domestic consumption safety, particularly for raw seafood. Given the limitations of traditional depuration methods, HPP, ozone treatment, and PL technology are emerging as future global standards for viral decontamination in seafood processing [27].

In many developing nations, enforcing strict seafood hygiene regulations presents significant challenges. Limited resources, inadequate infrastructure, and poor regulatory oversight contribute to increased risks of viral contamination [152]. Many seafood-producing regions rely on open-water aquaculture where wastewater contamination can introduce HAV and NoV into seafood stocks [153]. Additionally, lack of access to advanced processing technologies like HPP or PL means that traditional depuration and heat treatment remain the primary methods, despite their limited effectiveness against enteric viruses [27]. Inconsistent enforcement and limited training for food handlers further exacerbate safety risks [154]. Strengthening global collaborations, providing technical assistance, and improving sanitation infrastructure are crucial steps toward enhancing seafood safety in these regions [136].

### 3.3. Recent Viral Protection Technologies for the Seafood Industry

While we have gained considerable insights from past outbreaks, reducing the risk of outbreaks of traditional common viruses in the seafood supply chain by controlling pre-harvest contamination or vaccination has proven inadequate. It was only during the SARS-CoV-2 outbreak that we recognized the limitations of current technologies in addressing potential unknown viruses that may arise in the future. The mutation rate of SARS-CoV-2 is accelerating, and its transmission methods are diverse. To cope with the escalating complexity of emerging viruses in the future, more advanced technologies must be applied in the seafood industry.

The impact of the SARS-CoV-2 pandemic on the labor supply was significant, and any potential large-scale outbreak could cause a similar crisis. The food processing industry and fisheries production are labor-intensive and susceptible to short-term food supply disruptions due to local transportation and travel restrictions [155]. The implementation of Industry 4.0 technologies can provide better digital solutions in the food industry to prevent potential large-scale viral outbreaks and tackle manpower shortages [140]. Industry 4.0 is an intelligent advanced manufacturing and information technology system that provides real-time information in the production process through digital technologies such as artificial intelligence (AI) and the Internet of Things (IoT) to improve efficiency and reduce labor demand. Chitrakar [155] summarized several AI-based interventions aimed at reducing the impact of SARS-CoV-2 on the food supply chain, including intelligent modified atmosphere packaging technology with sensors, intelligent freezing and thawing technology for product preservation and processing, intelligent monitoring technology for food safety, and intelligent sensory inspection systems for real-time monitoring of food quality and freshness. Additionally, contactless intelligence is gaining attention in the retail sector at the end of supply chain. To some extent, smart devices, including those used for food delivery, have alleviated the impact of social restriction policies on the interests of catering service companies [156]. Contactless offline business models, such as unmanned stores and unmanned supermarkets, also contribute to more efficient consumer shopping, reducing administrative and labor costs while minimizing the risk of viral transmission [157]. Using advanced Industry 4.0 technologies, individuals can engage in remote employment with minimal labor demand, unaffected by restrictive rules [140].

As stated earlier, various viruses, whether enteric viruses or adenoviruses, including SARS-CoV-2, can attach to surfaces through food, food packaging materials, or worktops, posing a potential risk of contamination transmission. Developing new edible seafood packaging films and coatings may be an innovative technological direction to reduce the risk of foodborne infection transmission [158]. Dehghani [159] elaborated on the current research progress and applications of edible films in seafood, used for antioxidants, resistance to lipid and water loss, spoilage prevention, preservation, and antiviral properties. In the foreseeable future, advancements are anticipated in developing edible coatings with antiviral properties that can effectively maintain the freshness of fish and other seafood. The SARS-CoV-2 virus exhibits significant durability on common surface materials such as plastic and stainless steel, surviving for almost three days [21]. HAV and adenoviruses exhibit enhanced resilience on non-porous materials [137]. Silicon nitride (Si3N4), a novel bioceramic material, has shown significant potential in suppressing viral activity. Pezzotti [99] demonstrated that Si3N4 could inactivate 99% of the SARS-CoV-2 virus after just 1 min of exposure. Accordingly, the seafood industry could consider Si3N4 coatings as an alternative to other surface materials and incorporate Si3N4 particles into the manufacturing materials of PPE and work attire, while minimizing surface roughness to effectively mitigate viral attachment and transmission.

Furthermore, antiviral nanomaterials can help combat coronavirus infections transmitted through contaminated air and objects. Metal nanoparticles are currently used as antiviral drugs to develop new medical applications targeting various viruses. Metal nanoparticles can penetrate cell membranes, obstructing virus replication after attachment [160]. Although the significance of nanotechnology in combating coronaviruses has not been fully explored, it has shown potential in developing food packaging materials resistant to coronaviruses [155]. Polymers containing copper, silver, and zinc, along with nanostructures, can impede coronavirus contamination and transmission by inhibiting viral replication and other mechanisms. Among them, silver nanoparticles (AgNPs) engage with cell receptors, hindering the entry of HIV-1 virus into cells [160]. Similarly, iron oxide (ferric oxide) nanoparticles can interact with the S protein RBD of the SARS-CoV-2 virus, potentially preventing the virus’s attachment to host cells [161]. Metal (silver and gold) or metal oxide (copper, zinc, titanium, and iron oxide) nanoparticles have successfully reduced the infectivity of harmful viruses such as adenovirus and hepatitis virus [162]. AgNPs gold nanomaterials have shown efficacy against adenovirus type 3 at a concentration as low as 25 μg/mL, directly disrupting viral particles and DNA structures [163]. For norovirus, TiO2NPs can enhance inflammatory responses, thereby improving viral clearance [164]. AgNPs biosynthesized from safflower and yellow cress plant extracts have shown notable antiviral activity against HAV-10 [165]. This evidence suggests that nanomaterials may be a viable option for developing food packaging materials with antiviral properties [155]. Additionally, nanotechnology can be applied in anti-infective personal protective equipment (masks, face shields, head covers, and work clothes), textiles, surface coatings, and potent antiviral disinfectants [158,160]. Although there are still concerns about the possible adverse effects of nanoparticles on human health and environmental pollution, their low cost, high efficacy, and ease of synthesis are expected to reduce the impact of virus transmission in challenging environments and developing countries [166]. However, current antiviral active packaging materials face the issue of altering the physicochemical properties of food. This necessitates that the seafood industry and relevant researchers ensure that the development of antiviral packaging or edible film coatings does not compromise the inherent qualities of seafood.

The inactivation of viruses is commonly achieved through high temperatures; however, high temperature treatments are not widely applicable in the seafood industry. In recent years, there has been research into innovative non-thermal food processing technologies, including high-pressure processing (HPP), cold plasma (CP), ultraviolet light (UV), irradiation, and pulsed electric fields (PEF), aimed at enhancing food safety and reducing foodborne viruses. High-pressure processing, as a food preservation method, can inactivate microbes and enzymes, thus extending the shelf life of food while minimally impacting its sensory, physical, and nutritional properties [167]. Currently, the food industries in many countries have implemented HPP for various foods, including fresh shellfish, which are particularly prone to carrying viruses. It has been proven that HPP can inactivate noroviruses in shellfish [168]. Applications of HPP at ≤600 MPa have been shown to effectively inactivate a variety of non-enveloped and enteric viruses, including HAV and HEV, with a reduction of ≥5 log [168]. However, studies indicate that viruses in shellfish from natural environments exhibit higher resistance to HPP treatment compared to those cultivated in laboratories [169]. Research on ultraviolet light for food safety is extensive. Compared to thermal processing, applying UV technology to food results in minimally processed products with high freshness and minimal impact on product quality. UV irradiation inactivates microbes by causing lesions and hindering DNA replication. Recently, advances have been made in reducing noroviruses in contaminated oysters during the purification phase using filtration [170] and low-pressure UV (LP-UV) [31]. However, PEF has shown poor effectiveness in inactivating foodborne viruses; a study by Mohamed [171] demonstrated that various concentrations of rotavirus were not inactivated under PEF treatment at 20 to 29 kV/cm for 145.6 µs.

## 4. Conclusions

This paper elucidates the infection mechanisms and transmission routes of common viruses in the seafood supply chain, explaining the potential transmission threats posed by these viruses to the seafood industry and examining countermeasures. Despite the development of numerous strategies to reduce the incidence of outbreaks of historically prevalent viruses, such as norovirus, HAV, and HEV, our preparedness for the coronavirus outbreak was evidently insufficient, as judged from the results of this pandemic, resulting in a severe impact on the seafood supply chain. Although SARS-CoV-2 is no longer pandemic, as coronaviruses continue to mutate and evolve, they remain a potential threat to global seafood safety, either by undermining the integrity of the fisheries and aquaculture supply chains or by causing market volatility stemming from public mistrust in seafood safety. Despite many cases of frozen seafood testing positive for the virus, SARS-CoV-2 is classified as a respiratory virus rather than a foodborne virus. Consumers are unlikely to contract SARS-CoV-2 from the consumption of foods contaminated with the virus, which may alleviate some immediate negative perceptions. For the seafood industry, the best control strategy to prevent the transmission of future airborne pathogens, such as coronaviruses, within the supply chain is to eliminate opportunities for person-to-person transmission, as infected workers are the primary source of virus introduction. Nevertheless, implementing standard procedures to prevent the dissemination of foodborne illnesses remains essential, as these measures can better avoid outbreaks of traditionally common foodborne viruses, particularly norovirus, which dominates seafood-related viruses, and are also effective in preventing the transmission of airborne viruses like coronavirus. However, to revive the aquaculture industry in the future, there is a need for improved, more efficient, and safer food processing technologies, storage methods, and seafood supply chain management, requiring collaborative efforts from diverse stakeholders. The emergence of more virulent and transmissible coronaviruses during this SARS-CoV-2 outbreak, along with the sluggish advancement in treatment development, renders the delicate global seafood supply chain vulnerable to disruption. We should take inspiration from the outcomes of the SARS-CoV-2 outbreak to understand that the impact of viruses is long-term and prone to producing variants that pose new threats. Although the SARS-CoV-2 pandemic has passed, we may encounter other unknown viral outbreaks in the future; therefore, we must continue to monitor, assess, and document the impacts of each outbreak on the fisheries industry while considering appropriate responses to guide subsequent strategies and prepare for future pandemics. If the industry begins revitalizing the fisheries economy now, while also implementing more scientific management practices, building flexible supply chains, promoting new smart technologies, and leveraging marine resources and products, we are more likely to minimize losses in the next pandemic and better withstand any potential viral crisis.

## Figures and Tables

**Table 1:** Some food-borne viruses that are common in seafood.

Virus Common Name (Abbreviation and/Serotype)	Virus Family	Food Commodity	Causes of Diseases
Hepatitis A virus (HAV),	Picornaviridae	Bivalve molluscan shellfish (including oysters, clams, cockles, and mussels); fresh produce; prepared foods	Hepatitis
Norovirus (NoV),	Caliciviridae	Bivalve molluscan shellfish (including oysters, clams, cockles, and mussels); fresh produce; prepared foods	Gastroenteritis
Sapovirus	Caliciviridae	Salad; river water; oysters	Gastroenteritis
Enterovirus (e.g., poliovirus, Coxsackie A, B virus)	Picornaviridae	Oysters; contaminated water or food	Associated with a range of symptoms, including neurological symptoms
Hepatitis E virus (HEV)	Hepeviridae	Raw or undercooked meat of pig or wild boar or Sika deer; unpasteurized milk, shellfish, and ethnic foods; contaminated water	Hepatitis
Astrovirus	Astroviridae	Transmission is fecal–oral via food or water (<1% of astrovirus infections are considered food-borne) [25]	Gastroenteritis
Human parvovirus	Parvoviridae	Shellfish	Erythema infectiosum
Human adenovirus (HAdv)	Adenoviridae	Shellfish	Gastroenteritis
Hantavirus		Contamination of food or water with saliva or urine or through the dust of feces from infected wild rodents	Hantavirus pulmonary syndrome (HPS) and hemorrhagic fever with renal syndrome
Aichi virus	Picornaviridae	Oysters and seafood	Gastroenteritis

**Table 2:** Survivability of SARS-CoV-2 on different material surfaces at room temperature.

Environmental Conditions	Temperature	Viability Time
Aerosol	21–23 °C	Up to 3–4 h
Plastic	21–23 °C	Up to 72 h
Stainless steel	21–23 °C	Up to 72 h
Copper	21–23 °C	Up to 4 h
Cardboard	21–23 °C	Up to 24 h
Glass	21–23 °C	Up to 96 h
Cloth	22 °C	Up to 24 h
Steel	21–23 °C	Up to 48 h
Surgical mask	22 °C	Up to 96 h

**Table 3:** Surface disinfectant for potentially effective inactivation of SARS-CoV-2.

Disinfectants/Methods	Working Concentration or Condition	Treatment Time	Reduction of the Virus Titer	References
Heat treatment	>75 °C	45 s to 5 min	N/A	[98]
Sodium hypochlorite	0.05% (500 ppm)	5 min	SARS-CoV-2 reduced by about 3 logs	[98]
Silicon nitride	15 wt% aqueous suspensions	1 min	Inactivate 99% of SARS-CoV-2	[99]
Heat treatment	70 °C	5 min	SARS-CoV-2 reduced by about 7 logs	[73]
Ozone (O_3_)	4 ppm (gas exposure)	90 min	>98.2% viral titer reduction	[100]
Household bleach	1:49	5 min	SARS-CoV-2 reduced by about 5.8 log (>99.9%)	[73]
Household bleach	1:99 aq.	5 min	SARS-CoV-2 reduced by about 5.8 log (>99.9%)	[73]
Ethanol	70% aq.	5 min	SARS-CoV-2 reduced by about 5.8 log (>99.9%)	[73]
Povidone-iodine	7.5% aq.	5 min	SARS-CoV-2 reduced by about 3.8 log (>99.9%)	[73]
Chloroxylenol	0.05% aq.	5 min	SARS-CoV-2 reduced by about 4.8 log (>99.9%)	[73]
Chlorhexidine (0.05%)	0.05% aq.	5 min	SARS-CoV-2 reduced by about 3.8 log (>99.9%)	[73]
Benzalkonium chloride	0.1% aq.	5 min	SARS-CoV-2 reduced by about 3.8 log (>99.9%)	[73]

## Data Availability

The original contributions presented in the study are included in the article, further inquiries can be directed to the corresponding authors.
